# The relationship between olfaction and cognitive function in the elderly

**DOI:** 10.1186/s12576-020-00777-8

**Published:** 2020-10-14

**Authors:** Sae Uchida, Chiho Shimada, Naoko Sakuma, Fusako Kagitani, Akiko Kan, Shuichi Awata

**Affiliations:** 1 Department of Autonomic Neuroscience, Tokyo Metropolitan Institute of Gerontology, 35-2 Sakaecho, Itabashi-ku, Tokyo, 173-0015 Japan; 2 Department of Exploring End-of-Life Care for the Elderly, Tokyo Metropolitan Institute of Gerontology, Tokyo, Japan; 3 Department of Dementia and Mental Health Research, Tokyo Metropolitan Institute of Gerontology, Tokyo, Japan; 4 Department of Health Services Research, Tokyo Metropolitan Institute of Gerontology, Tokyo, Japan

**Keywords:** Odor identification threshold, Olfactory function, Cognitive function, Attention, Elderly people

## Abstract

This study investigated the relationship between olfaction and cognitive function in 12 elderly people (age: 80.9 ± 1.6) living in the community. Olfactory function was assessed by the identification threshold for rose odor. Four cognitive measures consisting general cognitive ability assessed by Mini-Mental State Examination (MMSE), its sub-domains, and attentional ability assessed by drawing a line to connect the numbers consecutively (trail-making test part A; TMT-A), were assessed. Subjects with a higher olfactory threshold (≥ 5) declined more in the performance speed of TMT-A (73% ± 7%, *p* = 0.05) compared with those subjects with a lower threshold (≤ 4) (averaged value was set at 100%). Other cognitive statuses assessed by MMSE tended to decline in subjects with higher thresholds. Because attentional function relates to the basal forebrain cholinergic system, our results suggest that olfactory impairment links to the decline in cognitive function, particularly of attention-relating cholinergic function.

## Introduction

The decline in olfactory function is one of the earliest symptoms of Alzheimer’s disease [1]. Difficulty in identifying odors predicts the subsequent transition from mild cognitive impairment to Alzheimer’s disease, and also from normal cognition to mild cognitive impairment [2, 3]. However, in these studies, they did not evaluate cognitive dysfunction, based on the levels of olfactory impairment. To establish the olfactory deficits as an early preclinical indicator for Alzheimer’s disease, on the basis of the level of olfactory impairment, decline in cognitive function should be evaluated, in the elderly people living in the community, using appropriate assessments.

The rose odor is one of the odorant items that has been reported to become harder to identify with the cognitive decline [4, 5].

Attentional ability, which relates to the basal forebrain cholinergic system [6, 7], is the first non-memory domain to be affected in Alzheimer’s disease [8]. Basic animal research has revealed that the activation of basal forebrain cholinergic neurons produces an increase in extracellular acetylcholine release in the neocortex, hippocampus, and olfactory bulb [9–15]. 

On the basis of these background studies, in early Alzheimer’s disease, we speculated that impairments of both the olfactory identification ability and the cognitive functions, especially attention, may arise from basal forebrain cholinergic deficits. This study aimed to investigate the relationship between olfactory identification ability and cognitive functions, including attention, in elderly people living in the community. In this study, the identification threshold for the rose odor was assessed as the olfactory identification ability. We analyzed four cognitive measures, including general cognitive ability assessed by the MMSE, its sub-domains, and attentional ability assessed by TMT-A.

## Methods

### Subjects

A total of 12 elderly people, aged 70–90 years (10 females and 2 males), living in the community, participated in this study. No one had a clinical diagnosis of dementia and a history of stroke. No one had nasal congestion or a runny nose on the day of examination.

The study was conducted under the declaration of Helsinki and approved by the Human Research Ethics Committee of the Tokyo Metropolitan Institute of Gerontology. Written informed consent was obtained from all the participants.

### Assessment of olfactory function

Olfactory ability was assessed by identification threshold for a rose odor by 2-phenylethyl alcohol (CAS no. 60-12-8; Tokyo Chemical Industry, Tokyo, Japan), which is a dominant odor compound in natural rose petals. A serial tenfold odorant dilution of eight steps [from 1 (low) to 8 (high) concentrations] was prepared with a starting concentration of 631 mg/ml. The odor solvent was diluted in propylene glycol (Maruishi Pharmaceutical Co., Ltd., Osaka, Japan) for the first three steps (step 6–8), and then in liquid paraffin (CAS no. 8042-47-5, Fujifilm Wako Chemicals Corporation, Osaka, Japan) in the later steps (step 1–5), according to the part of an olfactory sense testing device in Japan [16]. The subjects were presented with a 30 ml clear glass bottle containing a 200 µl odorant dilution-soaked paper and were asked to sniff the bottle twice. By way of a single ascending non-forced-choice method, the subjects detected the odor at a certain step and then identified the odor at the same step of detection or a higher concentration step [16]. In this study, we analyzed the lowest concentration step at which the subjects correctly identified the odor. When the subjects had difficulty in naming the odor, they were asked to recognize the odor by a card written with the correct odorant names (rose flower, faint sweet, flower, or plants). If no recognition (identification) was obtained, the higher concentration step was tested. The interval between steps was 20 s to avoid odor adaptation. The olfactory test took place in a quiet, well-ventilated room. A portable local ventilation equipment (SMST-DD-W-HD, Shonan Maruhachi S-Tech Co., Ltd., Kanagawa, Japan) set close to odorant bottles. Subjects were asked to avoid using perfume, and eating or drinking except water for more than 30 min before testing.

### Assessment of cognitive function

We assessed cognitive abilities consisting general cognitive ability (assessed by MMSE) [17], its sub-domains, and attentional ability assessed by TMT-A [18].

General cognitive ability was assessed using the Japanese revised-version of MMSE (Nihon Bunka Kagakusha, Tokyo, Japan). The MMSE contains multiple cognitive domains, including orientation, repetition, verbal recall, attention and calculation, language, and visual construction. The total scores for MMSE range from 0 to 30; scores 28–30 interpret as normal cognitive status, scores 24–27 interpret as mild cognitive impairment, and scores ≤ 23 interpret as suspected dementia [19, 20]. In addition to the total scores for general cognitive ability, we analyzed MMSE orientation and verbal recall 13-item subset, and 17 other items, as previously reported [4].

Attentional ability was assessed by TMT-A. The subjects were required to draw a continuous line to connect the encircled numbers of 1–25 consecutively, which are pseudo-randomly distributed on the sheet of paper used in our laboratory cohort study. The time needed to complete the task was measured. Before the test trial, a practice trial (1–8 numbers) was administered to make sure the subjects understand the task. All subjects had no visual deficit or motor problem that may disrupt the task performance.

MMSE and TMT-A were conducted by a clinical psychologist and researchers trained by the clinical psychologist, after the assessment of odor identification threshold on the day.

### Data analysis

The values were presented as means ± SEM, unless otherwise stated. Data analysis was done using Prism 5 (Graph-Pad Software Inc., San Diego, CA, USA). The Mann–Whitney test was used for comparison of four kinds of cognitive measures between two groups of different olfactory ability (low-threshold group vs. high-threshold group). A *p*-value ≤ 0.05 was considered to be statistically significant.

## Results

**Fig. 1:**
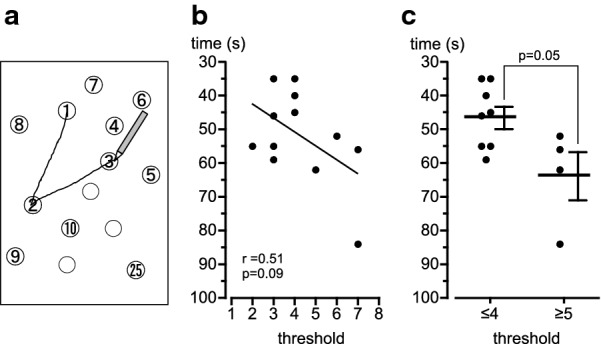
Relationship between attentional ability and odor identification threshold. **a** Method testing the attentional ability by drawing a line to connect the numbers (TMT-A). **b** Scatter plot of the relationship between attentional ability (time in s) and identification threshold for rose odor in all 12 subjects. The dots represent the values of an individual subject. Pearson's correlation coefficient *r* with *p*-value. **c** Comparison of attentional ability (time in seconds) between two groups of different olfactory ability, that is, low-threshold group (≤ 4) and high-threshold group (≥ 5). The horizontal lines and vertical bars show the mean and SEM values in each group. *p* = 0.05; the significant difference between low-threshold group (≤ 4) and high-threshold group (≥ 5) was tested by Mann–Whitney test

**Fig. 2:**
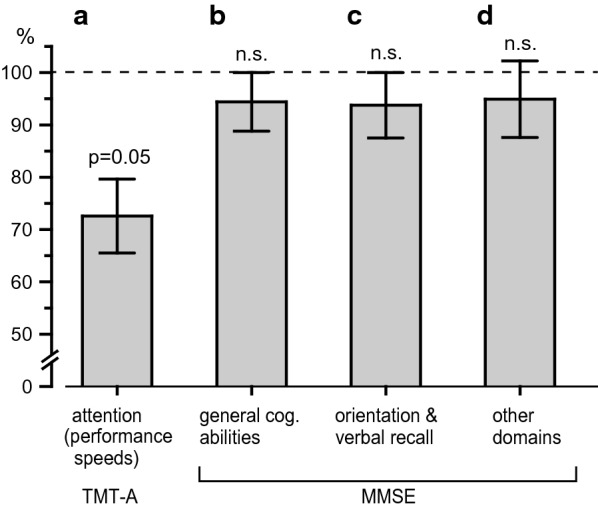
Comparisons of four cognitive measures in odor identification of high-threshold group. a: Attentional ability (performance speeds of TMT-A). b: General cognitive ability (MMSE, total score), c: orientation and verbal recall (MMSE, 13-item subset), d: other cognitive domains (MMSE, another 17-item subset). The averaged value was set at 100% in the low-threshold group (≤ 4), for each cognitive function. Columns a–d and vertical bars values were presented as means ± SEM in the high-threshold group (≥ 5)

As shown in Fig. 1a, the attentional ability was assessed by TMT-A, which is a test in drawing a line to connect the numbers from 1 to 25 consecutively. All subjects were able to complete the task, and the time needed to complete was 52.0 ± 3.9 s (from 35 to 84 s). Figure 1b shows the scatter plot of the relationship between attentional ability (vertical axis, the time needed to complete, TMT-A) and olfactory ability (horizontal axis, the threshold for identifying the rose odor) in all 12 subjects. All subjects were able to identify the odor between steps 2 and 7. The higher identification threshold showed a trend of longer time to complete the task (*r* = 0.51, *p* = 0.09). The scatter plot formed two clusters; left (*n* = 8), and right (*n* = 4). The left cluster tends to be low in odor identification threshold (threshold of steps 2–4) and fast in completing the task assessing attentional ability. The right cluster tends to be high in odor identification threshold (threshold of steps 5–7) and slow in completing the task assessing attentional ability. Therefore, in Fig. 1c, we compared the attentional ability between the low-threshold group (≤ 4; *n* = 8) and the high-threshold group (≥ 5; *n* = 4). The completion time of the task in the high-threshold group was 63.5 ± 7.1 s (median: 59.0), and the value was significantly higher than that in the low-threshold group (46.3 ± 3.3 s, median: 45.5) (*p* = 0.05). When the task performance speed in the low-threshold group was set at 100% as average, the performance speed in the high-threshold group was calculated to be 73% ± 7%, as shown in Fig. 2a.

In Fig. 2b–d, the other cognitive scores were analyzed in the same manner. General cognitive ability (MMSE, total score), orientation and verbal recall (MMSE, 13-item subset), and other cognitive domains (MMSE, another 17-item subset) in the high-threshold group were 94% ± 6%, 94% ± 6%, 95% ± 7%, respectively. There were tendencies in decline in these three cognitive measures in the high-threshold group, compared with that of the low-threshold group.

## Discussion

This study revealed the linking of olfactory impairment (increase in identification threshold for rose odor) to the decline in cognitive function, particularly of attention assessed by TMT-A, in elderly people living in the community.

In this study, all subjects could complete the task assessing attention (TMT-A) within the limit time (240 s). The slowest subject needed 84 s to complete the task. This value was much shorter than the limit time and was not an outlier (tested by Grubbs' test). The present study detected mild-to-moderate impairment of attention associating decline in olfactory identification ability in the elderly.

The attentional ability, which was assessed by TMT-A, is improved by the treatment of cholinesterase inhibitor in patients with mild Alzheimer’s disease [21]. Cholinergic neurons in the basal forebrain, which have a crucial role in attention, memory, and olfactory functions [6, 7, 22–25], undergo severe degeneration in patients with Alzheimer’s disease [26–28]. Therefore, it is speculated that olfactory impairment (increase in identification threshold for rose odor) linking to the decline in attentional ability may be relating to impairment of basal forebrain cholinergic function. Our results suggest that assessment of olfactory identification ability (threshold for rose odor) linking the attentional ability is useful for early detection of Alzheimer’s disease, which can be applied to the elderly especially having mild cognitive impairment.

In this study, general cognitive ability assessed by MMSE, and MMSE sub-domains (orientation and verbal recall MMSE 13-item subset, and remaining 17-item subset) tended to decline in subjects with higher olfactory threshold compared with subjects with a lower threshold. Umeda-Kameyama et al. [4] reported that declines in both the MMSE 13-item subset and the remaining 17-item subset correlating with the development of cognitive impairment (i.e., normal vs. mild cognitive impairment, and Alzheimer’s disease), although greater decline in the 13-item subset.

Pathological changes in Alzheimer’s disease, particularly neurofibrillary tangles, occur first in the olfactory information processing regions (olfactory bulb, anterior olfactory nucleus, entorhinal cortex, etc.) [1, 29, 30]. Rejis et al. [31] reported that lower odor identification scores were correlated with increased cerebrospinal fluid (CSF) total tau concentrations, but not with CSF amyloid-β (1–42) concentrations. Wilson et al. [32] suggested that the difficulty of older people in identifying odors is partly due to the accumulation of neurofibrillary pathological features within central olfactory pathways (e.g., entorhinal cortex and hippocampus). Tiernan et al. [33] suggested a link between tau pathology within cholinergic basal forebrain neurons and the progression of Alzheimer’s disease. Further studies are needed to clarify the relations of declines in olfactory identification, attention, and pathological brain features.

Olfactory stimulation has reported maintaining cholinergic innervation in the olfactory bulb, as well as increases acetylcholine release in the hippocampus [34, 35]. Olfactory stimulation such as smelling pleasant rose odor may be a useful intervention for the elderly preventing cognitive impairment.

The limitation of this study is the small sample size. Therefore, the results should be interpreted with caution. Additional studies with a larger sample size are recommended to verify the link between olfactory identification ability and attentive ability in the elderly. Odorants other than rose odor should also be tested for the identification ability in relation to cognitive function in the elderly. Also, the effects of possible confounding factors, such as age and education, should be clarified.

## Conclusion

This study shows that olfactory impairment assessed by the identification threshold for rose odor links to the decline in cognitive function, particularly attentive ability relating to brain cholinergic function, in elderly people living in the community.

## Data Availability

The data that support the findings of this study are available from the corresponding author on reasonable request.

## Abbreviations

CSF: Cerebrospinal fluid
MMSE: Mini-Mental State Examination
TMT-A: Trail-making test, part A
